# The Autophagy Machinery in Human-Parasitic Protists; Diverse Functions for Universally Conserved Proteins

**DOI:** 10.3390/cells10051258

**Published:** 2021-05-19

**Authors:** Hirokazu Sakamoto, Kumiko Nakada-Tsukui, Sébastien Besteiro

**Affiliations:** 1 Department of Infection and Host Defense, Graduate School of Medicine, Chiba University, Chiba 260-8670, Japan; 2 Department of Biomedical Chemistry, The University of Tokyo, Tokyo 113-8654, Japan; 3 Department of Parasitology, National Institute of Infectious Diseases, Tokyo 162-8640, Japan; kumiko@m.u-tokyo.ac.jp; 4 LPHI, Univ Montpellier, CNRS, INSERM, 34090 Montpellier, France

**Keywords:** autophagosome, *Trypanosoma*, *Leishmania*, *Entamoeba*, apicomplexa, apicoplast

## Abstract

Autophagy is a eukaryotic cellular machinery that is able to degrade large intracellular components, including organelles, and plays a pivotal role in cellular homeostasis. Target materials are enclosed by a double membrane vesicle called autophagosome, whose formation is coordinated by autophagy-related proteins (ATGs). Studies of yeast and Metazoa have identified approximately 40 ATGs. Genome projects for unicellular eukaryotes revealed that some ATGs are conserved in all eukaryotic supergroups but others have arisen or were lost during evolution in some specific lineages. In spite of an apparent reduction in the ATG molecular machinery found in parasitic protists, it has become clear that ATGs play an important role in stage differentiation or organelle maintenance, sometimes with an original function that is unrelated to canonical degradative autophagy. In this review, we aim to briefly summarize the current state of knowledge in parasitic protists, in the light of the latest important findings from more canonical model organisms. Determining the roles of ATGs and the diversity of their functions in various lineages is an important challenge for understanding the evolutionary background of autophagy.

## 1. Introduction

**Figure 1:**
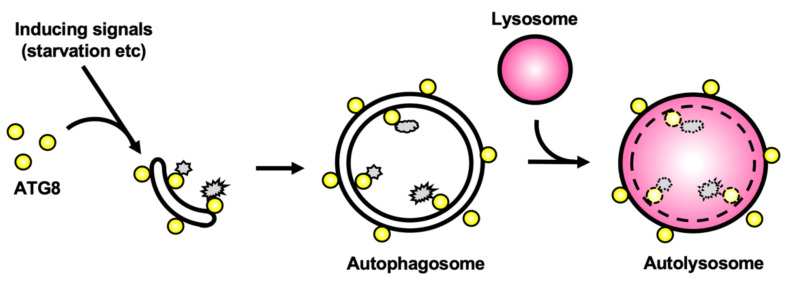
Schematic diagram of autophagy in mammals. Starvation and other stresses induce autophagy and ATG8-family proteins are recruited onto the autophagosome membrane. Proteins harboring an LC3-interacting region (LIR) motif can bind to ATG8-family proteins to be subsequently selectively degraded. Upon completion of the closure, autophagosomes and lysosomes fuse together to form the autolysosomes. The engulfed material (depicted in gray) is degraded and can be subsequently recycled. Notably, ATG8-family proteins are located both inside and outside the autolysosomes.

**Figure 2:**
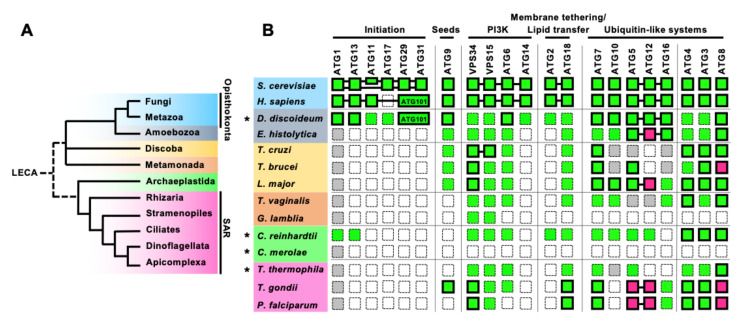
Conservation of the core ATGs in parasitic protists. (**A**) A simplified phylogenic tree of eukaryotes based on [21,22]. Fungi and Metazoa both belong to the Opisthokonta phylogenetic group. SAR includes Stramenopiles, Alveolata (Ciliates, Dinoflagellate, and Apicomplexa), and Rhizaria. (**B**) Conservation of the core ATG machinery in parasitic protists, compared with those of yeast, human, and free-living protists (shown by an asterisk). Conserved genes are shown in green and missing genes are shown in white. Genes with low overall homology to yeast Atgs, but with conserved specific motifs or domains are shown in gray. Magenta indicates the conservation of a functional homolog with atypical features. Proteins that have been functionally analyzed are enclosed by bold lines. The proteins with proven interactions are connected to each other by a line, including the atypical ATG12–ATG5 interaction characterized in Apicomplexa. *S. cerevisiae* has two scaffold proteins in the ATG1 complex, ATG11 and ATG17, while *H. sapiens* has one scaffold protein, FIP200 which is a homolog of ATG11. *H. sapiens* has ATG101 instead of ATG29 and ATG31. Conservation and function of ATGs in *Dictyostelium discoideum* are reviewed in [23,24], in [25] for *Chlamydomonas reinhardtii* (green algae) and *Cyanidioschyzon merolae* (red algae), and in [26] for *Tetrahymena thermophila*. Additional searches for members of the poorly conserved initiation complex, ATG11/FIP200, ATG17, and ATG29/ATG31 (ATG101), were conducted for *C. reinhardtii*, *C. merolae*, and *T. thermophila*, using human or yeast homologs as queries.

Macroautophagy, usually simply called ‘autophagy’, is a lysosomal degradation system of intracellular components mediated by double-membrane structures called autophagosomes (Figure 1). Autophagy has been characterized morphologically at the subcellular level by electron microscopy in the 1950s [1] (the history of autophagy is reviewed in [2,3]). However, the molecular mechanisms underlying autophagosome formation and the physiological significance of autophagy have remained major conundrums for a long time. In the 1990s, a seminal mutagenesis screen performed in *Saccharomyces cerevisiae* [4] has allowed the identification of genes coding for what is now referred to as autophagy-related (ATG) factors. Importantly, not only the repertoire of identified ATGs has expanded since then, but most homologs of the core ATG machinery were found to be universally conserved in mammals and other eukaryotes, albeit to a lesser extent in phylogenetically-distant organisms (Figure 2). Studies on model organisms have shown that autophagy is involved in a number of key cellular or physiological processes such as adaptation to nutrient starvation, senescence, organelle homeostasis, and immune mechanisms [5,6,7,8]. In particular, a large part of autophagy research has been focused on mammals, because deregulation of autophagy has been associated with several human diseases including cancer and neurodegeneration. However, the physiological significance of autophagy in phylogenetically-distant eukaryotes, like protists, is still poorly understood.

Protists are a heterogenous group of mostly unicellular eukaryotic organisms. Most protists are often difficult to cultivate or manipulate genetically, so they are not usually considered model organisms and are largely understudied. However, recent technical advances, such as single cell genomics, have allowed better characterization of marine protists, for example [9], and illustrated the tremendous evolutionary diversity of eukaryotes [10]. Not only protists can provide interesting insights into the cell biology of eukaryotes [11,12], but many are also responsible for important human diseases that affect millions of people around the world. As such, they are worthy subjects of research, and recent breakthroughs in genome editing techniques like CRISPR/Cas9 for example, now offer the prospect of tackling important biological questions in some of these organisms [13]. In this review, we summarize the current knowledge on the roles of the autophagy-related machinery in major human-parasitic protists.

We cover parasitic protists which are the most studied and thus usually those of high medical importance. One of the most investigated groups of parasites is the Trypanosomatidae, transmitted by insect vectors and responsible for major human diseases, such as leishmaniasis (caused by a variety of *Leishmania* species), African trypanosomiasis or sleeping sickness (caused by *Trypanosoma brucei*), and Chagas disease (caused by *T. cruzi*). Another group of relatively well-studied protists is Apicomplexa, a phylum containing parasites responsible for the deadly malaria (mainly caused by *Plasmodium falciparum*), or the more benign but largely ubiquitous toxoplasmosis (caused by *Toxoplasma gondii*). Less studied disease-causing protists include pathogenic Amoebozoa, such as dysentery-causing *Entamoeba histolytica*, or Metamonada which are responsible for intestinal (*Giardia lamblia*) or sexually-transmitted (*Trichomonas vaginalis*) diseases.

## 2. The ‘Core’ ATGs for Autophagosome Formation

To date, approximately 40 ATGs have been reported. Some of them have a specific function only in yeast or mammals, while about 20 ATGs, called the ‘core’ ATGs (Figure 2B), are considered to play similar roles in autophagy among most eukaryotes, including yeast, Metazoa, and land plants [14,15].

It should be noted that, traditionally, the nomenclature for autophagy-related protein names has differed between yeasts and other eukaryotes; ‘Atg’ is used for yeasts, and ‘ATG’ is used for Metazoa and plants. Some of them are also named differently in yeasts, mammals, and Nematoda, for instance Atg8 homologs (LC3, GABARAP, GATE16, or LGG). For the sake of simplicity, in this review, we unified the notation to ‘ATG’ in capital letters for protists and for other eukaryotes. Consequently, hereafter, we described ULKs as ATG1, BECN1, Vps30 as ATG6, WIPIs as ATG18, and so on, while ‘Atg’ is used to describe yeast proteins.

**Figure 3:**
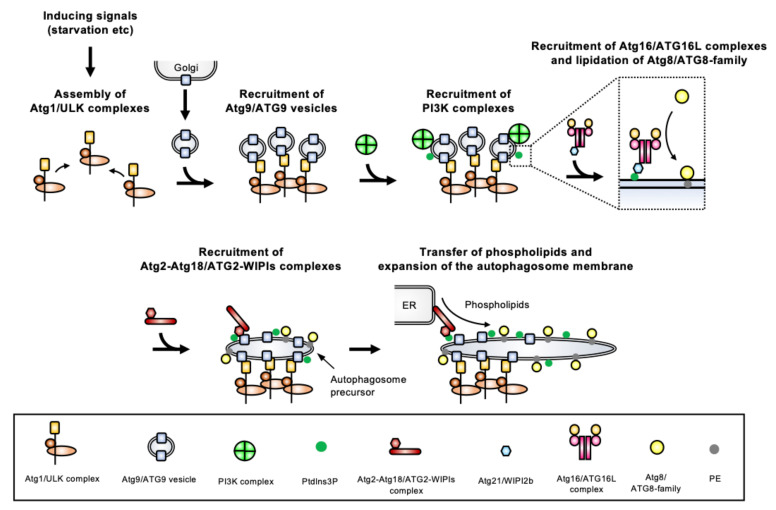
Schematic model for the molecular process of autophagosome biogenesis in Opisthokonta. Autophagy inducing signals, such as starvation, stimulate the assembly of the Atg1/ULK complex, which is a platform for initiating autophagosome formation. Then, Golgi apparatus-derived Atg9/ATG9 vesicles are recruited to the platform and may be seeds for autophagosome biogenesis. The PI3K complexes localize to this site after Atg9/ATG9 vesicles are recruited, and generate PtdIns3P. The PtdIns3P recruits the Atg16/ATG16L complexes to the vesicle depending on the PtdIns3P-binding activity of Atg21/WIPI2b, then proteins of the Atg8/ATG8 family are conjugated with PE on the vesicle. In addition, PtdIns3P recruits the Atg2-Atg18/ATG2-WIPIs complexes on the vesicles, and phospholipids are transferred to it from donor compartments such as ER by the lipid transfer activity of Atg2/ATG2. It enables the expansion of the autophagosome membrane. Since ATG8 homologs are localized on the autophagosome membrane from the beginning of its formation to the end of the degradation process, as shown in Figure 1, the protein is commonly used as a marker of autophagy.

These core ATGs are classified into six categories based on their roles: (1) the Atg1 complex (ULK (Unc-51-like kinase) complex in mammals), (2) the Atg9 vesicles, (3) the phosphatidylinositol 3-kinase (PI3K) complex, (4) the Atg2-Atg18 complex (ATG2-WIPI (WD-repeat protein interacting with phosphoinositides) complex in mammals), (5) the Atg12 conjugation system, and (6) the Atg8 conjugation system. Proteins from these six groups localize in a hierarchical manner to a pre-autophagosomal structure from which autophagosomes will be generated (Figure 3). The most complete information on the molecular process underlying autophagosome biogenesis has been obtained in Opisthokonta (mostly yeast and mammalian models, which are extensively studied). Here, we only provide a brief overview of the molecular machinery involved in autophagosome formation, as more detailed information is available elsewhere [5,14,15,16,17,18,19,20].

### 2.1. Atg1/ULK Complex

The Atg1 complex is composed of Atg1, Atg13, Atg17, Atg29, and Atg31 (and Atg11 for selective autophagy [27]) in yeast, and the mammalian ULK complex is composed of ULK1 or ULK2 (homologs of Atg1), ATG13, FAK family-interacting protein of 200 kDa (FIP200) (homolog of Atg11), and ATG101 (instead of Atg29 and Atg31). Atg17 and Atg11/FIP200 are scaffold proteins for the complex. Atg1/ULK is a kinase that is critical for initiation of autophagosome formation. During normal growth conditions, its activity is generally blocked by target of rapamycin complex 1 (TORC1), while starvation or some other stresses lead to the inhibition of the TORC1 function, allowing activation of the Atg1/ULK complex. The activated complex forms multimers via Atg13 which can crosslink two Atg17s [28], and liquid–liquid phase separation drives this assembly [16]. The assembled Atg1/ULK complex is a platform for initiation of autophagosome formation, which recruits other Atg/ATGs, and among them the Atg9/ATG9 vesicles.

### 2.2. Atg9/ATG9 Vesicles

The Atg9/ATG9 protein is integrated into membranes via its multiple-transmembrane regions (it is in fact the only transmembrane ATG identified to date). Atg9/ATG9 localizes on the Golgi apparatus, endosomes, or other membranes, but, importantly, it also localizes to distinct cytoplasmic *trans*-Golgi network-derived vesicles called the Atg9/ATG9 vesicles [29]. During starvation, these vesicles are recruited to the assembled Atg1/ULK complex. Since the Atg9/ATG9 vesicles mainly localize at the site of early autophagosome formation, they are likely involved in autophagosome nucleation. In fact, an excellent in vitro reconstruction of the yeast autophagy system has demonstrated that Atg9 vesicles are seeds for autophagosome biogenesis. They initiate the transfer of lipids from donor compartments such as the ER [30], and the recently demonstrated lipid scrambling activity of yeast Atg9 and human ATG9 suggests this protein is likely involved in lipid redistribution between membranes [31,32].

### 2.3. PI3K Complex

The PI3K protein complex includes vacuolar-protein-sorting (Vps) 34, Vps15, Atg6 (also known as Vps30), and Atg14 in yeast, and VPS34, p150 (homolog of Vps15), Beclin-1 (BECN1) (homolog of Atg6), and ATG14L in mammals. The class III PI3K Vps34/VPS34 produces phosphatidylinositol 3-phosphate (PtdIns3P) via phosphorylation of PtdIns. This complex localizes to the site of autophagosome formation after the Atg9/ATG9 vesicles have been recruited. However, how the PI3K complex and its product PtdIns3P are targeted to the autophagosome membrane is still unclear. The Vps34/VPS34 kinase is also involved in cellular functions other than autophagy through interactions with different partners, but only the Atg14/ATG14L-containing PI3K complex is involved in autophagy. The PtdIns3P produced by the PI3K complex recruits Atg18/WIPI to the autophagosome membrane.

### 2.4. Atg2-Atg18/ATG2-WIPI Complex

The PROPPIN (β-propellers that bind polyphosphoinositides) family includes Atg18 in yeast and four WIPI proteins (WIPI1-4) in mammals, which are PtdIns3P-binding proteins. These PROPPINs form a complex with Atg2/ATG2 that is recruited to autophagosome membranes depending on the PtdIns3P-binding capacity of the PROPPINs. Atg2/ATG2 is a multifunctional protein involved in membrane tethering and in a lipid transfer activity that was recently demonstrated in yeasts [30,33] and mammals [34,35].

### 2.5. Atg12/ATG12 Conjugation System (Atg16/ATG16L Complex)

**Figure 4:**
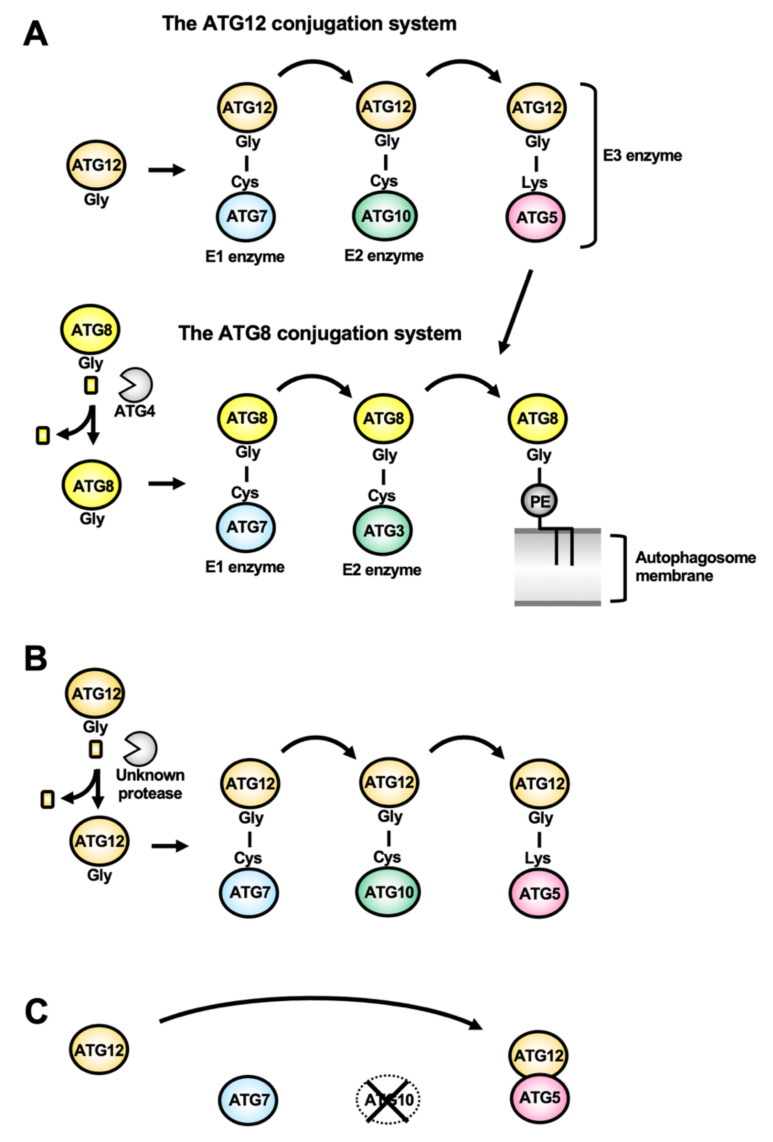
The two ubiquitin-like conjugation systems in autophagy and unique features of the ATG12 system in parasitic protists. (**A**) The ubiquitin-like reactions of ATG12 and ATG8 conjugation as found in most eukaryotes. A ubiquitin-like protein, ATG12, conjugates with ATG5 between the Gly residue at its most C-terminal in ATG12, and the acceptor Lys residue in ATG5. This is catalyzed by ATG7 (E1 enzyme) and ATG10 (E2 enzyme). ATG8, which is also a ubiquitin-like protein, undergoes a C-terminal processing by the ATG4 protease to expose a Gly residue. ATG8 can then be conjugated with the membrane-embedded lipid phosphatidylethanolamine (PE). This is catalyzed by ATG7 (E1 enzyme), ATG3 (E2 enzyme), and the ATG12–ATG5 conjugate (E3 enzyme). (**B**) The ATG12 conjugation system in *Leishmania major*. LmATG12 has a C-terminal tail like ATG8 homologs. It was demonstrated that a recombinant LmATG12 terminating with a Gly can conjugate with LmATG5. However, the protease involved in the processing of LmATG12 has not been identified. (**C**) The non-covalent ATG12-ATG5 complex in Apicomplexa. Apicomplexan parasites lost the ubiquitin-like reactions for ATG12. They do not have a ATG10 homolog and their ATG12 homolog has lost the C-terminal Gly residue. Instead, apicomplexan ATG12 forms a complex with ATG5 via a non-covalent interaction, and the complex acts as the E3 enzyme for ATG8 lipidation.

Atg12/ATG12 is a ubiquitin-like protein whose C-terminal Gly residue is conjugating to an acceptor Lys residue in Atg5/ATG5 (Figure 4A). The Atg12–Atg5/ATG12–ATG5 conjugate is formed thanks to the Atg7/ATG7 E1 enzyme and the Atg10/ATG10 E2 enzyme. The conjugate binds to Atg16/ATG16L, forming the so-called Atg16/ATG16L complex, that forms a dimer. The Atg16/ATG16L complex is recruited on the autophagosome membrane depending on the PtdIns3P-binding activity of Atg21/WIPI2b. The Atg16/ATG16L complex acts as the E3 enzyme for Atg8/ATG8-family proteins.

### 2.6. Atg8/ATG8-Family Conjugation System

Atg8 in yeast, and ATG8-family proteins (ATG8s, comprising proteins of the LC3 and GATE-16/GABARAP sub-families) in mammals, are ubiquitin-like proteins [36]. After processing of the C-terminal tails in ATG8s by the cysteine protease Atg4/ATG4, their C-terminal Gly residue is exposed, allowing conjugation with the phosphatidylethanolamine (PE) lipid present in the expanding autophagosome membrane. Atg4/ATG4 processing thus allows lipid conjugation to happen, but this protease is also involved in Atg8/ATG8 recycling from the membrane, making it an important regulator of the autophagic function [37]. This Atg8/ATG8s-PE conjugate is formed thanks to the E1 enzyme Atg7/ATG7, the E2 enzyme Atg3/ATG3, and the E3 enzyme Atg5-Atg12/ATG12-ATG5 (Figure 4A). The lipid conjugation of Atg8/ATG8s is the final step of autophagosome biogenesis and is required for the expansion of the autophagosome membrane. In addition, because of their association not only with nascent autophagosomes, but also their persistence on mature autophagic vesicles, Atg8/ATG8s have been used as common molecular markers of autophagy in many eukaryotes [38,39,40,41,42].

## 3. Overview of Conservation of the Core ATGs in Protists

Protists represent a large part of global eukaryotic biodiversity, in terms of lineages, morphology, but also molecular divergence. Here, we provide an updated overview of the conservation of ATGs in parasitic protists (Figure 2B), which has been summarized in several previously published reviews [43,44,45]. Additionally, updated reviews have been published for specific protist lineages, whether they are parasitic like Apicomplexa [46,47], or free-living like photosynthetic algae [25], Ciliates [26], and Amoebozoa (*Dictyostelium discoideum*) [24]. Overall, the presence of partially-conserved molecular machinery in these very phylogenetically diverse eukaryotes, suggests parts of the machinery may have been present already in the last eukaryotic common ancestor (LECA) [48]. However, there is a strikingly reduced ATG repertoire in most parasitic protists (*Giardia* being the most extreme with no obvious ATG conserved).

Streamlining of some features in some protists may reflect an adaptation to the parasitic lifestyle, which is, for instance, potentially illustrated by a reduced ATG repertoire in the parasitic amoeba *E. histolytica*, compared with the free-living amoeba *D. discoideum.* Yet, it seems more generally that protists, which are phylogenetically distant from Opisthokonta (comprising yeasts and mammals, in which most ATGs have been identified), clearly do not express the full complement of ATG homologs. One extreme example is the free-living red alga *Cyanidioschyzon merolae*, which does not seem to have any of the core ATGs [49]. Thus, although this might reflect a complexification of the machinery in Opisthokonta, there were also probably some secondary losses in protist lineages.

Strikingly, the most conserved part of the machinery is the Atg8/ATG8s membrane conjugation system; on the other hand, the nearly complete absence of the protein complexes for initiating autophagosome formation in parasitic protists raises the question as to how they regulate and induce autophagy.

### 3.1. Poor Conservation of the Early Components of the Autophagy Machinery in Protists

In many protists, the ATG1 complex is extremely poorly conserved, and ATG13 and ATG17 homologs are not found, in particular in parasitic protists [50]. Homology searches using ScAtg1 as a query hit some proteins homologous to the kinase domain, but these lack other regions involved in regulation and protein-binding. It is not known whether the ATG1 complex has emerged in only some supergroups, or if it has been lost secondarily in many protists. The pathogenic amoeba *Entamoeba*, for instance, has probably lost the complex, since its close phylogenetic relative *Dictyostelium* conserves all components of the ATG1 complex. ATG9 is conserved in Trypanosomatidae and Amoebozoa, but only partially conserved in Apicomplexa (it is only present in Coccidia, like *Toxoplasma* for instance), and is seemingly absent from Ciliates [26].

### 3.2. Conservation of the PtdIns3P Signaling Complex

VPS34, VPS15, and ATG6 (VPS30) are universally conserved in protists, but not ATG14. It should be noted that these conserved proteins composing the PI3K complex are involved not only in autophagy but also in other cellular functions, like endosome formation and vesicular trafficking, in yeast and mammals [51,52]. Therefore, whether they are involved in autophagy to the same extent in protists is still an important open question.

ATG18, which binds to VPS34/PI3K-generated PtdIns3P, is generally conserved in protists, whereas its partner ATG2, a lipid transfer protein important for autophagosome membrane expansion, is missing. Homology searches using ScAtg2 as a query retrieve several protist proteins with homology to its ATG_C domain (pfam09333 entry in the Pfam 34.0 database. Available online: http://pfam.xfam.org/, accessed on 19 May 2021), but they usually do not possess the Chorein_N domain (pfam12624) responsible for lipid transfer to the autophagosome membrane [33,34,35]. Thus, most parasitic protists seem not to have functional homologs of ATG2, suggesting that other lineage-specific molecular mechanisms may substitute for it.

### 3.3. The ATG8 Conjugation System Is Conserved in Almost All Protists

The most conserved part of the ATG machinery in parasitic protists is clearly the ATG8 membrane conjugation system. The ATG8 family proteins are not only the most widely used autophagy markers in eukaryotes, but also key proteins for the formation of autophagosomes. Consequently, ATG8 homologs are probably the most studied ATGs in protists, as illustrated by the following section.

## 4. Autophagy in Parasitic Protists

### 4.1. Trypanosomatidae

#### 4.1.1. The ATG8 Conjugation System: ATG8s Are Multicopy and Some of Them Are Atypical

**Figure 5:**
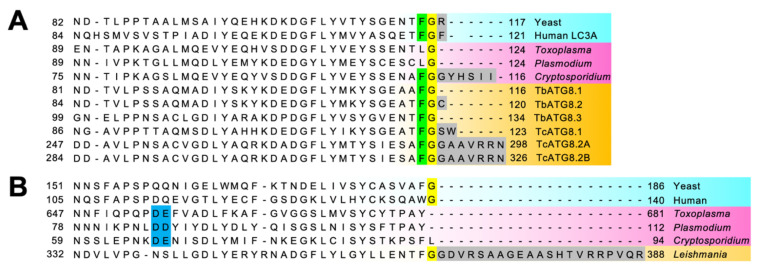
Sequence alignment of the C-terminal regions of ATG8 and ATG12 homologs from selected protists and human and yeast counterparts. (**A**) Sequences of ATG8 homologs from *S. cerevisiae* (Yeast), *H. sapiens* (Human LC3A), *T. gondii* (TGGT1_254120), *P. falciparum* (PF3D7_1019900), *C. parvum* (XP_001388400.1), *T. brucei* TbATG8.1 (Tb927.7.5900), TbATG8.2 (Tb927.7.5910), TbATG8.3 (Tb927.7.3320), *T. cruzi* TcATG8.1 (TcCLB.508173.47), TcATG8.2 (TcCLB.510533.180), TcATG8.2b (TcCLB.511821.160) are shown. The Gly residue that is responsible for conjugation with PE is highlighted in yellow. Green and gray highlight the highly conserved aromatic amino acids before one of the Gly and the C-terminal extension after the Gly, respectively. (**B**) Sequences of ATG12 homologs from *S. cerevisiae* (Yeast), *H. sapiens* (Human LC3A), *T. gondii* (TGGT1_321300), *P. falciparum* (PF3D7_1470000), *C. parvum* (cgd7_3873), *L. major* (LmjF22.1300) are shown. The Gly residue that is responsible for conjugation with ATG5 is highlighted in yellow. Blue and gray highlight positively charged amino acids that are critical for the interaction with ATG5 in Apicomplexa and the C-terminal extension after the Gly, respectively.

ATGs involved in the ATG8 conjugation system (ATG8, ATG7, ATG3, and ATG4) are conserved in *T. brucei*, *T. cruzi*, and *L. major*. Noticeably, the ATG8-like protein family is expanded in Trypanosomatidae: three ATG8s in *T. brucei* [44] and *T. cruzi* [53], and up to 25 ATG8s in *L. major* [54]. For instance, in *L. major*, the 25 ATG8 homologs can be classified into four types: 2 LmATG8, 3 LmATG8A, 9 LmATG8B, and 13 LmATG8C [55]. Although these isoforms appear to have some functional overlap, this diversification may reflect some differences in their physiological role. Another peculiarity is that, unlike the typical eukaryotic ATG8 homologs, TbATG8.1 and TbATG8.3 end with a Gly residue (Figure 5A), as for apicomplexan ATG8s (see Section 4.2.4 below), which may have implication in the regulation of their membrane association.

#### 4.1.2. The ATG12 Conjugation System: Originality and Uncertainty

ATGs involved in the ATG12 conjugation system (ATG12, ATG10, and ATG5) are conserved in *L. major* [54], and their ubiquitin-like conjugation reaction has been experimentally determined [56]. Unlike typical ATG12 homologs, LmATG12 does not end with a Gly residue, but has a 10-residue extension like typical ATG8 homologs (Figure 5B). Although peptidases that may process this extension are still unknown, a recombinant LmATG12 ending with a Gly residue forms a conjugate with LmATG5 [56] (Figure 4B) and compensates for autophagy flux in *atg12* knockout (KO) yeasts [54]. As for other Trypanosomatidae, it is still unclear if *T. brucei* and *T. cruzi* have a fully conserved ATG12-dependent conjugation system and some researchers presume that it has been completely lost in *T. cruzi* [57]. On the other hand, while conservation of ATG12 has not been demonstrated, ATG5 is found in *T. brucei* and is involved in autophagy as described below [58]. Like ATG8, ATG12 is a ubiquitin-like protein, and it should be noted that TbATG8.3 and TcATG8.2 might be functional homologs of ATG12 instead, as they are syntenic with LmATG12 and they do not associate to autophagosome-like vesicles (discussed below) [53,58].

#### 4.1.3. Autophagosome-Like Vesicles in Trypanosomatidae

In *T. brucei*, starvation conditions induce double-membrane autophagosome-like vesicles, which have been characterized using electron microscopy (EM), and were shown by immuno-cryoEM experiments to contain TbATG8.2 [59]. Immunofluorescence analyses have also shown that starvation induces the formation of TbATG8.1- and TbATG8.2-labelled puncta, but not TbATG8.3 [58,59], and this is inhibited by knockdown of *TbATG7*, *TbATG3*, and *TbATG5* [58].

In *T. cruzi*, double-membrane autophagosome-like vesicles are found in the parasite upon anti-trypanosome drug treatments [60,61]. Yet, only the TcATG8.1 isoform was experimentally confirmed to be involved autophagy. It conjugates with PE and localizes on the autophagosome-like vesicles upon starvation [53]. Treatment with wortmannin, an autophagy inhibitor, impairs the formation of TcATG8.1-positive vesicles during starvation [62].

Autophagosome-like vesicles have been observed by EM in some *Leishmania* spp. under treatment with anti-parasitic drugs [63,64] and under starvation conditions [65,66]. Overall, canonical autophagic vesicles seem to be induced by starvation and other stimuli in a regulated fashion in these Trypanosomatidae parasites.

#### 4.1.4. Physiological Significance of the ATGs in the Life Cycle of Trypanosomatidae

The overall contribution of autophagy to parasite physiology may be different between the different developmental stages of *T. brucei* [58]. While disrupting autophagy (through *TbATG7* or *TbATG5* knockdown) did not lead to detectable phenotypes in cellular proliferation or differentiation of the mammalian stages, inactivating the pathway (through *TbATG7* or *TbATG3* knockdown) diminishes proliferation of the insect stages under starvation.

The function and physiological significance of autophagy in *T. cruzi* was recently reviewed in [57]. It has been known that in vitro-applied nutritional stress that mimicked conditions encountered in the insect vector can trigger metacyclogenesis (differentiation from epimastigotes to metacyclic trypomastigotes). Interestingly, in the insect forms of *T. cruzi*, wortmannin treatment not only impairs the formation of TcATG8.1-positive vesicles during starvation, but also decreases metacyclogenesis, showing that starvation-induced autophagy is involved in metacyclogenesis [62].

In *L. major*, the LmATG8-positive puncta increase accompanies differentiation to infective metacyclic promastigotes from promastigotes (metacyclogenesis, occurring in the insect vector) and also in amastigotes (the developmental form found in mammalian cells) [55], suggesting autophagy may play a role in the process. In fact, autophagy-deficient mutants were used to show that autophagy is critical for metacyclogenesis; differentiation, when monitored using a metacyclic-marker [67], is impacted in both the *LmATG5* KO [56] and *LmATG4.2* KO [66] parasites. However, disrupting the function of another ATG4 homolog, LmATG4.1, does not seem to affect autophagy [68], which might reflect some functional diversification for ATG proteins.

#### 4.1.5. Autophagy in Organelle Turnover

Autophagy may also be involved in organelle turnover in Trypanosomatidae. These parasites compartmentalize glycolytic enzymes into peroxisome-related organelles called glycosomes [69,70]. In the mammalian stage parasites, ATP generation depends essentially on glycolysis. In contrast, to adapt to nutrient sources present in the host, the insect stage parasites switch to other pathways for energy production: preferentially using proline and threonine through the tricarboxylic acid cycle and the electron transport chain located in mitochondria to generate ATP. This metabolic change implies remodeling of the glycosomal components and turnover of this peroxisome-related organelle in the transition from mammalian to insect stages. On the other hand, it is known that peroxisome turnover in other organisms is mediated by a selective autophagic process called pexophagy [71,72], and a similar mechanism may be at play for glycosome turnover [69]. In both the insect and mammalian stages of *Leishmania* spp, knocking-out *LmATG5* leads to an increase in the number of glycosomes; moreover, LmATG8 localizes to the glycosomes and also to the lysosomes, suggesting autophagosome-surrounded glycosomes are degraded by lysosomes [73]. In *T. brucei*, colocalization between lysosomal and glycosomal markers is increased during differentiation [74], although the direct involvement of TbATG8 and of the rest of the core ATG machinery in organelle turnover was not clearly demonstrated. It was shown that TbATG5 is dispensable for mammalian to insect stage differentiation [58], but glycosomes were not evaluated in that context. Thus, it is possible that glycosome turnover may not be essential for the differentiation process, or that some ATG-independent autophagy, as known to happen in yeast, mammals, and plants [75,76,77], may be involved in that particular context.

Other peculiar organelles, called reservosomes, are observed specifically in the insect stages of *T. cruzi*. They are lysosome-related organelles in which epimastigotes (found in the insect host) store proteins and lipids uptaken by endocytosis [78]. The stored materials are degraded by cruzipain (cruzain), a papain-like cathepsin L-like cysteine protease [79], and this is critical for the differentiation process [80]. Reservosomes disappear following the degradation of their contents. Notably, starvation-induced autophagy leads to the activation of cruzipain [81], and reservosomal components are consumed, which is essential for parasite survival and for differentiation in the insect stages of *T. cruzi*.

In Trypanosomatidae, which have a single mitochondrion, autophagy is also potentially involved in mitochondrial homeostasis. For instance, in *LmATG5* KO parasites, the mitochondrial mass increases and the membrane potential is decreased during starvation, reflecting non-functional organelles and/or degradation [56]. In wild-type cells, EM analysis shows that starvation induces fragmentation of the mitochondrion [73]. H_2_O_2_ treatment can also induce mitochondrial fragmentation, and LmATG8 was found to localize on both mitochondria and lysosomes in these conditions [73]. This suggests that upon stress the damaged mitochondrion fragments can be degraded by autophagy in these parasites.

The implications of proteolytic activity and lysosome-autophagosome fusion for autophagy have been shown in *Leishmania* spp. The importance of proteases for *Leishmania* spp. survival has been demonstrated (reviewed in [55]). Double-KO of Cathepsin L-like cysteine proteases *CPA* and *CPB* (the main lysosomal proteases) inhibits metacyclogenesis and transformation to amastigotes, and ATG8-positive puncta accumulate in amastigotes [65]. This suggests that the autophagy flux is arrested due to lysosomal dysfunction. The endosomal sorting complex required for transport (ESCRT) machinery has been shown in fly, nematode and mammalian cells to be involved in forming multivesicular bodies (MVB), but also in the autophagosome closure (reviewed in [82]). VPS4, an ATPase, is involved in completing the ESCRT-mediated membrane remodeling. Overexpression of ATPase-defective mutant LmVPS4 leads to the accumulation of LmATG8-positive puncta [66], suggesting that that functional MVBs are needed for lysosome-autophagosome fusion in *Leishmania*.

#### 4.1.6. Alternative Ways of Regulating Autophagy in Trypanosomatidae?

Trypanosomatidae do not appear to have a conserved ATG1 complex pathway for initiation of autophagy, but may involve original ways of regulating the formation and maturation of autophagic vesicles. For instance, one study has proposed that initiation of autophagy in *T. brucei* upon starvation requires the acidification of acidocalcisomes [83], which are lysosome-related organelles [84].

On the other hand, the involvement of the PI3K complex in autophagy has been verified and seems more canonical, like in *T. cruzi*. As mentioned above, wortmannin, an inhibitor for the PI3K activity of VPS34, impairs the starvation-induced autophagosome formation in *T. cruzi* [62]. Another study also demonstrates the importance of the kinase activity of TcVPS34: like in other eukaryotes, TcVPS15 interacts with TcVPS34 and regulates its kinase activity, and this complex localizes on TcATG8.1-positive vesicles in starvation conditions [85]. However, the importance for autophagy of other proteins, potentially also part of the PI3K complex, is still unclear.

### 4.2. Apicomplexa

#### 4.2.1. ATG8 Has Atypical Features in Apicomplexa

The updated summary of ATGs conservation in *P. falciparum* and *T. gondii* (as well as other Apicomplexa) is shown in [46,47]. ATG8 homologs can be found across all genera in Apicomplexa, but they are usually atypical: the C-terminal tail after the Gly (used for conjugation to PE) and the aromatic amino acid before the Gly are not present. It should be noted that the ortholog from *Cryptosporidium*, however, still bears typical ATG8 features (Figure 5A). The atypical ATG8 does not need processing by ATG4 prior to its lipidation [86,87], but instead ATG4 seems to play a role only in delipidation/recycling of ATG8 from membranes [88].

#### 4.2.2. An Atypical ATG12-ATG5 “Complex”: The First Evidence for Evolutionary Loss of Covalent Conjugation in Ubiquitin-Like ATG12

Another peculiarity in Apicomplexa, like *Plasmodium* and *Toxoplasma*, is that both the C-terminal Gly residues in ATG12 (the residue for covalent conjugation with ATG5) and ATG10 (an enzyme for catalyzing formation of the ATG12–ATG5 conjugate) are missing, even though they possess an ATG5 homolog (Figure 2B). In general, the ubiquitin-like conjugation reaction for formation of ATG12–ATG5 conjugate is essential for autophagy, especially for ATG8 lipidation [20]. However, apicomplexan ATG12 can interact with ATG5 without the ubiquitin-like conjugation reaction, and this ‘ATG12-ATG5 complex’ has the original E3 activity for ATG8 lipidation (Figure 4C) [87]. This is the first report of the evolutionary transition from ubiquitin-like conjugation to non-covalent interaction. This evolutionary innovation is important from an energetic point of view, because the ubiquitin(-like) conjugation system requires the E1-E3 proteins and ATPs to form the covalent conjugation. This raises questions as to why many organisms have retained the system for ATG12–ATG5 covalent conjugation while the non-covalent ATG12-ATG5 complex would be sufficient for the E3 function.

In other Apicomplexa, like *Eimeria* and piroplasms (*Babesia* and *Theileria*), ATG12 and ATG5 are simply not found [87]. Based on phylogenic relationships, these losses of the ATG12 system appear to have occurred independently. These ATG12-less parasites still conserve the ATG8 system, but it is unclear how they would regulate the lipidation and localization of ATG8.

#### 4.2.3. Autophagosome-Like Vesicles in Apicomplexa

Double- or multiple-membrane autophagosome-like vesicles have been observed using EM analysis in *T. gondii* [89,90,91,92] and *Plasmodium* spp [93,94]. ATG8-decorated autophagosome-like vesicles were also shown in *T. gondii* by immuno-EM [89].

Some studies suggest that autophagy is involved in organelle clearance during *Plasmodium* liver stage differentiation [95,96,97]. However, no lysosome-like compartment has been characterized in this stage, therefore how the organelle is degraded is a critical outstanding question. Importantly, Voss et al. [97] have shown that the autophagosome-like ATG8-positive vesicles colocalize with Golgi reassembly and stacking protein (GRASP)- and VPS4-containing vesicles that are involved in formation of the amphisome (an autophagic vacuole formed by fusion of an autophagosome and an endosome) [98]. They suggested that ATG8-positive vesicles can fuse with amphisomes and then release their content into the extracellular space in *Plasmodium* liver stages instead of going through an intracellular degradation compartment.

In extracellular *T. gondii* tachyzoites (fast replicating stages responsible for the acute phase of toxoplasmosis), TgATG8-positive puncta are induced by amino-acid starvation [88,89] and ER stress (both for intracellular and extracellular parasites) [92]. Degradation of autophagosome-contained material likely happens in a lysosome-like organelle called the vacuolar compartment (VAC) [99,100], which contains cathepsin B and cathepsin L proteases [101]. In *T. gondii*, canonical degradative autophagy seems to be involved for surviving stress conditions encountered when parasites are extracellular, but also in the context of the mammalian host, as supported by functional analyses of a TgATG9 mutant [102]. In the chronic stage parasites (bradyzoites), cathepsin L-depleted cells accumulate TgATG8-positive puncta, suggesting an arrest of autophagy [103]. The recycling of amino acids by autophagy may be crucial for the persistence of *Toxoplasma* in its host, as demonstrated by specific inactivation of ATG9 in this stage [104]. While Apicomplexa of the coccidia subclass (including *Toxoplasma*) possess an ATG9 homolog that seems essential for canonical autophagy akin to other eukaryotic models, other Apicomplexa like *Plasmodium* do not, which raises the question of how autophagosomes can be generated in these parasites.

#### 4.2.4. ATGs Are Essential for Apicoplast Biogenesis

An unexpected feature of apicomplexan ATGs is that some are involved in the maintenance of the apicoplast, a four-membrane plastid derived by secondary endosymbiosis that hosts essential metabolic pathways [105]. Some immuno-EM analyses have shown that ATG8 also localizes on the apicoplast [86,96], and many fluorescence microscopic analyses have demonstrated that ATG8 co-localizes with apicoplast markers both in *Plasmodium* and *Toxoplasma* (reviewed in [106]). Today, the apicoplast localization of ATG8 is widely accepted, and the implication of VPS34 [107,108], ATG18 [109,110], and ATG4 [88] for apicoplast biogenesis has also been demonstrated. Moreover, a mutagenesis screening identified PfATG7 as an essential factor for apicoplast biogenesis [111]. Early actors of autophagosome biogenesis such as TgATG9, however, do not participate in this [102]. Instead, it is mostly the machinery regulating ATG8 membrane association that has this additional apicoplast-related function. When the parasites are in stress conditions apicomplexan ATG8 decorates autophagosomes, yet in normal growth conditions it is constitutively lipidated and localizes on the outermost membrane of the apicoplast. Unlike other ATG8 eukaryotic homologs, because of its role in maintaining the homeostasis of a vital organelle, apicomplexan ATG8 is thus essential for survival even in normal growing conditions. For instance, conditional knockdown approaches for *P. falciparum* and *T. gondii* show that apicomplexan ATG8 is critical for the apicoplast inheritance into daughter cells and the viability of the parasites [112,113]. On the other hand, it is interesting to notice that some Apicomplexa, like *Cryptosporidium* spp, which have lost the apicoplast during evolution, possess an ATG8 homolog with typical C-terminal tail features (Figure 5A). As a result of this, *Cryptosporidium* would be a key organism for investigation into canonical apicomplexan autophagy.

### 4.3. Entamoeba

#### 4.3.1. ATG12 and ATG8 Conjugation Systems in *Entamoeba* spp

All ATGs for the ATG8 conjugation system are identified in *Entamoeba* spp, but the conservation of ATG12, ATG10, and ATG16 remained unclear in *E. histolytica*. Intriguingly, however, *E. histolytica* bears a potential ATG5 homolog containing a canonical acceptor Lys residue for conjugation with ATG12. Interestingly, among EhATG5 partners we have recently identified by co-immunoprecipitation and mass spectrometry, we found an atypical EhATG12 and a WD domain-harboring EhATG16 (Nakada-Tsukui et al., unpublished). Although further characterization of the ATG12 conjugation system is needed in *E. histolytica*, this shows that experimental investigations can reveal unexpected features of the autophagy machinery in protists otherwise masked by an apparent absence of homology with canonical counterparts.

#### 4.3.2. Function of the ATGs in *Entamoeba* spp

In *E. histolytica*, autophagosome-like vacuoles have been observed under treatment with a plant alkaloid, berberine sulfate [114]. However, starvation-induced autophagy has not been demonstrated so far. In addition, serum and glucose starvation do not cause significant change of EhATG8 localization (Nakada-Tsukui, unpublished observation). EhATG8 is constitutively lipidated and localizes on the phagocytic cup at an early step of phagocytosis [115], while newly identified EhATG12 does not localize on it (Nakada-Tsukui et al., unpublished). Thus, it is currently unclear how EhATG8 is recruited onto the phagosomes. Knockdown of *EhATG8* impairs acidification of phagosomes and endosomes [115]. This is independent of the already known acidification pathway via EhRab7A and EhVps26 [116]. In another *Entamoeba*, reptilian parasite *E. invadens*, the number of EiATG8-positive puncta is increased during trophozoite development and encystation during normal growth condition, but not during amino acids starvation [117]. However, 1:1-diluted glucose-free medium can induce encystation of *E. invadens*, indicating energy starvation and low osmotic pressure may induce autophagy. Notably, in another amoeba genus, *Acanthamoeba castellanii*, knockdown of *AcATG12* or *AcATG16* reduced cyst formation, showing autophagy could be involved in encystation [118,119]. Taken together, it seems autophagy in *Entamoeba* is constantly activated and may function primarily in the uptake of extracellular materials in trophozoites and turnover of intercellular components to provide an energy source for encystation.

### 4.4. Metamonada (Giardia, Trichomonas)

#### 4.4.1. The ATG Machinery in Metamonada

All ATGs for the ATG8 conjugation system are identified in *T. vaginalis*, whereas ATGs for the ATG12 conjugation system are unclear. The parasite has two ATG8 isoforms [120,121]. In stark contrast, for *G. lamblia* all the main ATGs except for VPS34 and Vps15 homologs (also involved in non-autophagic processes) are missing, suggesting a complete absence of autophagy in this pathogen with very streamlined metabolic features (for instance, *Giardia* does not have classical lysosomes [122]).

#### 4.4.2. Function of the ATGs in Metamonada

An autophagosome-like vacuole has been reported under treatment with berberine sulfate [114] or under iron- and glucose-restricted conditions [121] in *T. vaginalis*. Expression of genes encoding TvATG4 and TvATG8a is upregulated by incubation in glucose-free medium [123]. In addition, TvATG8a- and TvATG8b-positive puncta increased upon glucose starvation, and the puncta formation was inhibited by treating with the VPS34 inhibitor wortmannin [120] and was accumulated by E-64d (a lysosomal protease inhibitor) treatment [121]. In glucose-rich conditions, autophagy was apparently stimulated by proteasome inhibition and led to the degradation of poly-ubiquitinated proteins [120]. Overall, this suggests that the ATG machinery is involved in a canonical proteolytic function in *T. vaginalis*.

## 5. Conclusions

Most parasitic protists have complex life cycles, often implying they have to undergo significant morphological changes between developmental stages, and also that they are likely to encounter different environmental conditions. Beyond morphological observations of vesicles resembling autophagosomes, demonstration of a fully functional autophagy pathway in parasitic protists is sometimes not firmly established, as several models are understudied or less amenable to cell culture or genetic manipulation. However, in the best studied groups of parasites (Trypanosomatidae and Apicomplexa), converging evidence shows that canonical degradative autophagy is involved in the response to stress, organelle turnover, or homeostasis, and as such is likely important for stage differentiation or adaptation to a changing environment. However, it should be noted that the degradation of the autophagocytosed material is often not demonstrated in parasitic protists. It would thus be particularly important to develop and adapt autophagy probes and monitoring assays that are useful in other eukaryotic systems to not only detect autophagy, but also to measure the autophagic flux [39,40,41] and lysosomal activity [124].

A puzzling observation is that the core ATGs show various degrees of conservation in parasitic protists, raising questions as to how the formation of autophagosomes is regulated. In particular, the absence of the ATG1 complex, of ATG2, and sometimes ATG9. By analogy with classical eukaryotic models for autophagy, these proteins are needed to provide the starting point for autophagosome formation, and its expansion [30,31,32,33,34,35]. However, most studies on autophagosome formation have been performed on phylogenetically-related yeast and mammalian models. It is thus possible that more divergent lineages have evolved specific machineries different from those of the Opisthokonta. An ATG1-independent induction of autophagy has been recently demonstrated in plants for instance [125]. Identifying early autophagy regulators in protists represents a considerable challenge but will be rewarding as it may also bring information about alternate pathways potentially also present in other more classical eukaryotic models. To clarify autophagosome biogenesis in protists, it will be important to analyze, in particular, the involvement of ATG2, ATG9, and other lipid transfer proteins involved in liquid–liquid phase separation that regulates the formation of the autophagosome [30,31,32,33,34,35,126]. In vitro reconstitution systems, as used recently to demonstrate the non-covalent ATG12-ATG5 complex and its E3 function in Apicomplexa [87], may also be valuable tools to study the autophagy machinery in some protists for which genetic manipulation or in vivo studies are difficult.

Although autophagy is likely a very ancient process whose primary function in nutrient and stress adaptation seems widely conserved in eukaryotes, more and more diverse and unconventional functions for ATGs are now being revealed. For instance, the implication of ATG8 homologs and their membrane conjugation machinery in a variety of non-autophagic dynamic membrane events such as LC3-associated phagocytosis (LAP), trafficking, or secretion and exocytosis, are now a widely demonstrated [127]. In this context, the discovery that ATG8 can regulate apicoplast inheritance in apicomplexan parasites is, for example, particularly interesting. As specialized forms of autophagy and LAP are used by mammalian cells to eliminate microbial pathogens, the presence of ATG8 on the outermost membrane (likely of phagocytic origin) of an endosymbiotic organelle to control its division may not be completely fortuitous and establishes an interesting parallel. If anything, it also illustrates that the tremendous diversity of protists provides a unique opportunity to look into eukaryotic adaptations and innovations. This definitely calls for further investigations of the autophagy pathway in these fascinating organisms.

## Data Availability

Data sharing not applicable.
